# Enhancing Analytical Sensitivity and Selectivity for Methylene Blue Determination in Water Samples by Using Multiphase Electroextraction Coupled with Optical Absorption Spectroscopy and Surface-Enhanced Raman Scattering

**DOI:** 10.1021/acsomega.4c03125

**Published:** 2024-07-20

**Authors:** Tarlene P. Miranda, Ricardo M. Orlando, Cristiano Fantini, Mariana R. Almeida

**Affiliations:** † Departamento de Química, Instituto de Ciências Exatas, Universidade Federal de Minas Gerais, UFMG, Belo Horizonte, MG 31270-901, Brazil; ‡ Laboratório de Microfluídica e Separações, LaMS, Departamento de Química, Universidade Federal de Minas Gerais, Belo Horizonte, Minas Gerais 30123-970, Brazil; § Departamento de Física, Instituto de Ciências Exatas, Universidade Federal de Minas Gerais, UFMG, Belo Horizonte, MG 31270-901, Brazil

## Abstract

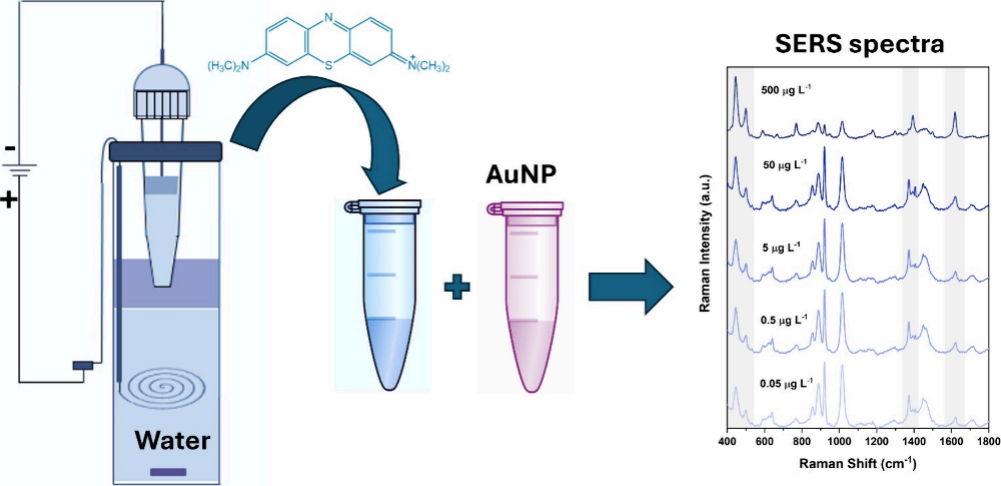

While optical analysis spectroscopy offers operational ease and low cost, it suffers from limitations regarding sensitivity when it comes to analyzing analytes at low concentrations. On the other hand, surface-enhanced Raman spectroscopy (SERS) offers high sensitivity but low selectivity in complex matrices. In this study, we have effectively addressed these challenges by integrating multiphase electroextraction (MPEE) as a sample preparation technique with these two spectroscopic methods for determining methylene blue (MB) dye in tap water samples. A Box-Behnken design was utilized for optimizing electroextraction parameters such as extraction time, pH, and acetonitrile percentage in the donor phase. After optimization, optical absorption spectroscopy results in a linear analytical curve within the range of 30 to 375 mg L^–1^ of MB, with method validation demonstrating high precision (relative standard deviation between 3.0 and 9.9%), recovery (99–105%), and detection and quantification limits of 1.3 and 4.0 μg L^–1^, respectively. On the other hand, using SERS, it was possible to detect MB in concentrations as low as 0.05 μg L^–1^. The extremely low concentrations of MB detected (in the range of a few ppb and ppt) and the acceptable validation performance parameters obtained highlight the potential of MPEE to enhance the applicability of spectroscopic techniques in routine analyses, especially when dealing with complex and challenging samples.

## 1 Introduction

Ensuring the quality of water for consumption poses an extremely challenging task for analytical chemistry, given the vast number of potentially harmful compounds present.^1−3^ Their concentrations are typically in the low parts per billion (ppb), parts per trillion (ppt), or even parts per quadrillion (ppq) range, and the samples are considered complex and rich in interferents. Reference techniques based on chromatographic systems (gas and liquid) coupled with state-of-the-art mass spectrometers are undoubtedly the choice to address this challenge and obtain the desired results.^4^ However, it is widely acknowledged that these instruments can cost over a million dollars for acquisition, require highly specialized expertise, and entail costly and periodic maintenance for proper operation.

In this context, spectroscopic techniques can become a viable alternative for specific analyses or in locations lacking access to sophisticated equipment. For instance, Raman spectroscopy has proven to be a promising and powerful option for the detection of organic molecules.^5^ Through surface-enhanced Raman scattering (SERS) strategies, it becomes possible to detect organic molecules at concentrations as low as individual molecules. SERS is a phenomenon in which the Raman scattering intensity of molecules is significantly enhanced when they come into contact with nanostructured metal surfaces. This enhancement primarily arises from three main sources: electromagnetic enhancement (EM), which amplifies the incident and scattered electromagnetic fields due to surface plasmon resonance; chemical enhancement, involving alterations in a molecule’s chemical properties when adsorbed on a metal surface; and geometric enhancement, where surface nanostructures concentrate molecules in high electromagnetic field regions, increasing the likelihood of Raman scattering. These combined effects make SERS a powerful tool for ultrasensitive molecular detection.^6,7^

While the SERS effect provides high sensitivity in noncomplex samples, its detection capability and selectivity become compromised in complex samples, thereby constraining the practical utility of this technique. Typically, complex samples containing trace-level analytes necessitate intensive presample preparation to eliminate interferents and concentrate the species of interest. Consequently, sample preparation plays a significant role in enhancing the sensitivity and selectivity of an analysis.^4,8^

In the past two decades, there has been significant diversification in sample preparation techniques, with new modalities emerging each year to make this step faster, safer, more efficient, and environmentally friendly.^9−11^ Electroextraction in porous hollow fiber membranes, named electromembrane extraction (EME), is one of these techniques that has gained widespread use and has been applied for different matrices (biological, environmental, forensic, etc.) and classes of analytes (drugs, metals, pollutants, pesticides, peptides, proteins, etc.).^12^ The general principle of electroextraction and the primary techniques derived from it involve the selective migration of electrically non-neutral analytes from a donor phase (sample) to an electrolyte solution (acceptor phase) through an organic filter that separates these two phases.^13^ Two major advantages are obtained with this strategy. First, essentially only species with an electric charge opposite to that of the electrode in the acceptor phase will efficiently reach the acceptor phase, providing high selectivity to the process. The second advantage is that, by using small volumes of the acceptor phase, a significant preconcentration can be achieved. In addition to these features, the vast majority of techniques based on electric fields offer superior speed, high automation capacity, batch extraction, and low consumption of reagents and solvents.^13−17^

After the publication of the first article describing EME, several new electroextraction modalities and approaches have been developed, with dozens of articles being published each year, all aimed at further enhancing the qualities above-mentioned.^12^ A more recent development in the electroextraction technique introduced a solid support into the acceptor phase in an approach known as multiphase electroextraction (MPEE).^18^ This strategy has opened new possibilities for sample preparation, as this solid material, after capturing the analytes, allows for their storage, transport, desorption for subsequent analysis,^19,20^ image capture for digital image analysis,^18,21^ or even coupling to mass spectrometry for direct analysis.^22,23^

Considering the continual advancements in this field of research, this study introduces, for the first time, the combination MPEE with optical absorption spectroscopy and SERS. Optical absorption spectroscopy was employed as a cost-effective and reliable technique for method development and validation, while SERS was utilized under optimized MPEE conditions to showcase its potential in achieving remarkably low concentration levels without significant challenges. Both spectroscopic techniques were applied in conjunction with MPEE to quantify the presence of MB in tap water. Methylene blue, a cationic thiazine dye, finds diverse applications (biological staining, the photographic industry, fish medicine, textile pigmentation, etc.) and has been detected in various water samples.^24^ It is well established that MB exhibits significant toxicity to both humans and animals.^25^ Consequently, the development of rapid, straightforward, and cost-effective analytical methods for the detection and quantification of MB in tap water holds paramount importance for water quality control assessment.

## 2 Experimental Section

### 2.1 Chemicals and Materials

Methylene blue, glacial acetic acid (HAc), citric acid, sodium phosphate, and ethanol were obtained from Synth (Diadema, SP, Brazil). Acetonitrile (ACN), methanol (MEOH), and ethanol (ETOH), all HPLC grade, as well as hydrochloric acid, were sourced from Merck (Darmstadt, Germany). 1-Octanol was purchased from Neon (Suzano, SP, Brazil), while 2-ethylxanole was acquired from Sigma-Aldrich (Darmstadt, Germany). All reagents used were of analytical purity or higher. Ultrapure water was obtained from a Milli-Q Simplicity 185 purification system (Millipore, Molsheim, France). Hydrophilic cotton wool (Apolo, Brazil) and stainless-steel wool (Scotch Brite, Brazil) were procured from a local supermarket, and conventional polypropylene blue micropipette tips were obtained from Kasvi (China). The electrodes were constructed from stainless-steel wire (0.4 mm i.d., SteelMesh, Tatuape, Brazil), and conventional polypropylene tubes with a capacity of 50 mL (Kasvi, China) were used for extractions.

### 2.2 Solutions and Samples

The MB stock solution was prepared in ultrapure water at a concentration of 1000 mg L^–1^. A McIlvaine buffer solution was prepared following the procedure described previously.^26^ A mixture of methanol, acetonitrile, and acetic acid was prepared in the proportions of 2:2:1 (v/v/v) (MEOH:ACN:HAc) for desorption of the sorbent material in the acceptor phase.

Fresh tap water samples were collected after 2 min of flushing from a single tap in the laboratory (Laboratório de Microfluídica e Separações, LaMS, at the Chemistry Department–UFMG–Belo Horizonte, Brazil). The donor phase (32.0 mL) consisted of a mixture of tap water samples and McIlvaine buffer (pH adjusted and described in the following sections) in a 5:1 (v/v) ratio, spiked with MB. The pH of buffer solutions was measured using a pH meter (HI2221, HANNA Instruments).

### 2.3 Large-Volume Multiphase Electroextraction Setup

**Figure 1:**
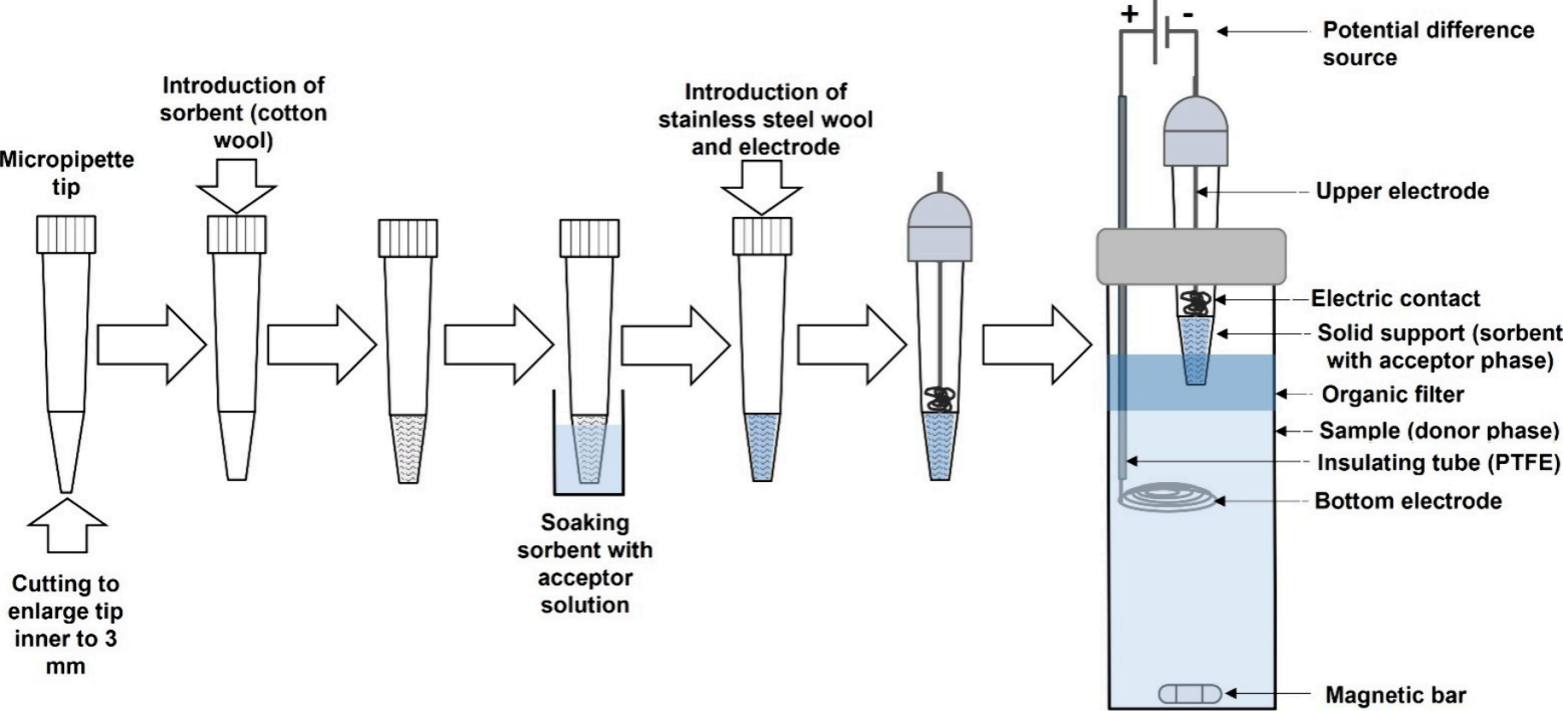
Schematic of the multiphase electroextraction system for electroextraction of MB from water samples.

A multiphase electroextraction system with capacity up to 10 extractions simultaneously was used.^20^ To set up the extraction unit, the 50 mL tubes were filled with 32 mL of the donor phase and then with 3 mL of immiscible organic filter (1-octanol). The sorbent material (0.03 g of cotton wool) was previously soaked in a 15 μL aqueous electrolyte solution and kept in contact with 0.05 g of stainless-steel wool. After that, the set composed of the micropipette with a sorbent, acceptor solution, and electrode were introduced as described in Figure 1.

A potential of 300 V was applied between the donor and acceptor phases using an electrophoresis source (model K33–300 V, Kasvi, China), and the electric current was monitored with a multimeter (model TP4000ZC, Tekpower, Montclair, CA, USA) during the extractions.

### 2.4 Electroextraction Optimization by Multivariate Approaches

The first step of the study involved screening and selecting the most significant variables using a 2^6–3^ fractional factorial design. Six variables (type of acceptor phase electrolyte, donor phase pH, type of organic solvent in the donor phase, extraction time, sorbent amount in the acceptor phase, and type of organic filter), all of which have been previously reported in the literature and considered determinants in the migration process of several other analytes during extractions with the application of an electric field,^13^ were examined at two levels, as shown in Table 1.

**Table 1:** Variables Investigated in the 2^6–3^ Fractional Factorial Design

	level	
variable	**–1**	**+1**
type of acceptor phase electrolyte (pH at 2.5)	HAc, pH = 2.50	HCl, pH = 2.50
donor phase pH	2.00	7.00
type of organic solvent in the donor phase (20%, v/v)	EtOH	ACN
extraction time (min)	10	20
sorbent amount in the acceptor phase (g)	0.0150	0.0300
type of organic filter	2-ethylhexanol	1-octanol

Following the selection of the variables extraction time, donor phase pH, and type of organic solvent in the donor phase, a Box-Behnken response surface methodology was employed to optimize these three parameters. With three significant variables, as detailed in Table 2, a total of 15 experiments were conducted, with three replicates at the central point.

**Table 2:** Variables Optimized in the Box-Behnken Design

	level		
variable	****–1****	**0**	**1**
extraction time (min.)	15	20	25
donor phase pH	5.00	6.00	7.00
% (v/v) of organic solvent (ACN) in the donor phase	30	35	40

The water samples were spiked with 4.0 and 0.5 mg L^–1^ MB for the 2^6–3^ fractional factorial and Box-Behnken designs, respectively. Electroextractions were performed using the free organic filter system, without agitation, and the donor phase was directly prepared in McIlvaine buffer, considering the pH values from the planning matrix. The design matrices for both the 2^6–3^ fractional factorial and Box-Behnken designs are presented in Tables S1 and S2 in the Supporting Information.

After extraction, MB was desorbed from the sorbent by transferring the cotton wool to a polypropylene microtube, adding a 860 μL MeOH:ACN:HAc solution (described in Section 2.2), and agitating it with a vortex for 30 s at 2000 rpm. The optical absorbance signals from the desorption solutions, as described in Section 2.5, served as the analytical response, and the experimental results were analyzed using Design Expert v. Eleven software.

### 2.5 UV–Vis Optical Absorption Analysis

The electronic spectra of the extracts were carried out in a UV–vis spectrometer Cary 60 (Agilent) in the range of 400–800 nm with a spectral resolution of 1 nm and an optical path of 1 cm. The UV–vis spectrum was used during the optimization and validation method. All MB quantifications were carried out using the absorption data obtained at 654 nm.

### 2.6 Validation Parameters

After establishing the optimal conditions for the electroextraction of MB, the following validation parameters were assessed: linearity (0.030, 0.075, 0.150, 0.225, 0.300, and 0.375 mg L^–1^, in triplicate each in spiked tap water); matrix effect (same range of linearity but using deionized water); intraday precision, interday precision (on two different days), and accuracy in terms of recovery (eq S1, Figure S2), all using spiked tap water at 0.030, 0.225, and 0.375 mg L^–1^ in sextuplicate (*n* = 6). Extraction efficiency (eq S2, Figure S2), limit of quantification (LOQ) (eq S3, Figure S2), and limit of detection (LOD) (eq S4, Figure S2) were determined using a UV–vis spectrometer as the analytical technique for the desorptions from the extractions.

The conditions for the electroextractions were as follows: the donor phase consisted of 32.0 mL of a mixture of tap water: McIlvaine buffer (pH = 5.00) with 35% ACN (v/v); acceptor phase electrolyte: 0.50 mol L^–1^ HAc solution; solid support of the acceptor phase: 0.0300 g of cotton wool; electric potential: 300 V; potential application time: 25 min; organic filter: 3 mL of 1-octanol; tip diameter of the probe: 3 mm.

### 2.7 Preconcentration Factor

The preconcentration factor (PF) under the optimized electroextraction conditions was calculated. To achieve this, six spiked water samples containing MB at a concentration of 0.030 mg L^–1^ were prepared (*n* = 6). Subsequently, the preconcentration factor (PF) (eq S5, Figure S2) was determined by calculating the ratio between the concentration of MB in the donor phase before extraction (*C*_ap_) and the concentration of MB in the desorption solution (*C*_dp_).

### 2.8 SERS Analysis

To obtain the SERS spectra, a Raman Horiba T64000 spectrometer was used, with monochromator mode, 600 lines/mm grating, excitation at 785 nm of a Ti:sapphire laser, 30 mW of laser power on the sample, 50× objective and 5 accumulations of 60 s, and a spectral resolution of 1 cm^–1^. Data acquisition was performed using Labspec 6 software, and the spectra were treated using the Origin 2018 program.

Five water samples spiked with MB solutions were prepared ranging from 500 to 0.05 μg L^–1^, and then electroextractions under optimized conditions were performed. A 150 μL desorption solution from electroextraction, a 200 μL suspension of gold nanoparticles (AuNPs), and 30 μL of 0.10 mol L^–1^ NaCl solution were added. Au colloidal nanoparticles (AuNPs) were prepared using a procedure described in a previous work.^27^ The description of the synthesis and characterization of the AuNPs used to obtain the SERS spectrum are included in the Supporting Information. After preparing this mixture, homogenization was carried out and 10 min was waited before taking the readings on the equipment.

## 3 Results and Discussion

### 3.1 Electroextraction Optimization by Multivariate Approaches

**Figure 2:**
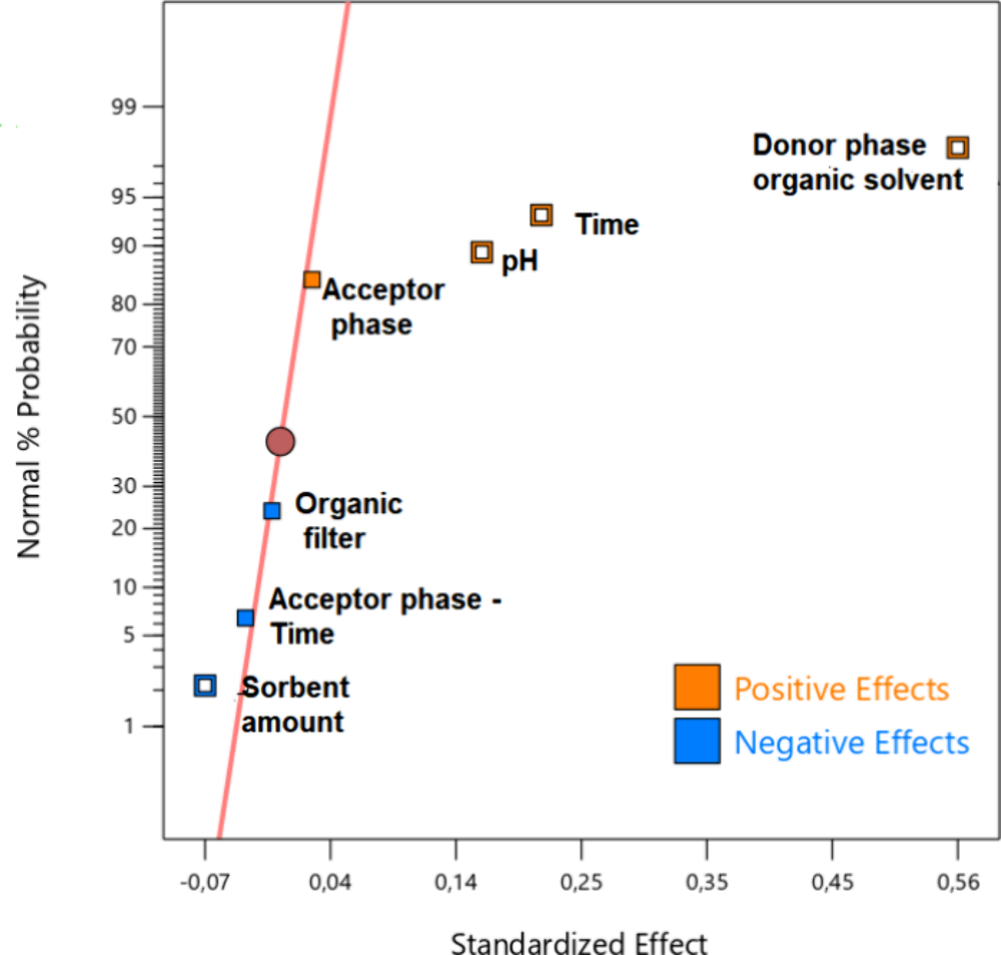
Normal probability plot of the estimate effects for 2^6–3^ fractional factorial design. Conditions: electric potential difference of 300 V; MB in the donor phase at 4.0 mg L^–1^.

The results of the fractional factorial design are depicted in Figure 2, revealing the significant variables for the electroextraction process in order of importance: the type of organic solvent present in the donor phase, extraction time, and the pH of the donor phase.

All significant factors exhibited positive effects, meaning that an increase in the levels of these variables leads to higher response values, in this case, absorbance, indicating enhanced extraction. The addition of ACN, a water-miscible organic solvent, can be employed to intensify the effect of the electric field on the donor phase (DP) and to reduce the interfacial tension between the DP and OF (organic filter).^28^ Extraction time is also known to be crucial for the transfer of analytes from the DP to the AP (acceptor phase), according to theoretical models.^29^ On the other hand, the pH of the DP can exert both positive and negative influences, depending on changes in analyte ionization and ion concentration at the DP-OF interface, as well as the formation of a capacitive ionic barrier.^30^

The nonsignificant effects follow a normal distribution and are on the line, with the main effects being the AP and OF. The nonsignificant effects conform to a normal distribution and include those found along the baseline, with the primary factors being AP and OF. Although AP has been previously described as an important parameter in other studies and has been suggested to influence ion-pairing effects,^31^ this parameter did not exhibit significant effects in our study. Between the linear (1-octanol) and branched (2-ethylhexanol) chain alcohols, we anticipated similar results as observed in Figure 2, given their closely matched physical and chemical properties. Furthermore, our research demonstrated that these two organic phases can be mutually substituted without substantial losses. Finally, increasing the amount of adsorbent had a negative effect. The negative effect on the amount of adsorbent can be explained by the increase in electric resistance conferred by the growth of the column of sorbent material by increasing its mass, thus decreasing the efficiency of electroextraction.

Based on these results, the selected variables for an optimization design included ACN as the organic solvent in the donor phase, extraction time, and pH of the donor phase. Meanwhile, OF and AP were held constant during the optimization process. 1-Octanol was chosen as OF due to its economic accessibility and lower toxicity profile. Acetic acid was selected as the acceptor phase electrolyte, as extractions using HCl led to slight heating. Last, the quantity of adsorbent, though not highly significant, was set at its maximum level, even with a negative effect, to ensure an ample supply of sorbent material under conditions of high extraction in the optimization design.

After initiating the preliminary tests for conducting the Box-Behnken optimization design, the concentration of the donor phase was reduced from 4.0 to 0.5 mg L^–1^ to prevent saturation of the sorbent during electroextraction. This reduction was implemented due to the significant increase in dye extraction observed under the new conditions. For a statistical assessment of the influence of the selected factors (% of ACN added to DP, pH of DP, and extraction time), a model was fitted using the least-squares method for the absorbance data, yielding the results presented in Table S3.

**Figure 3:**
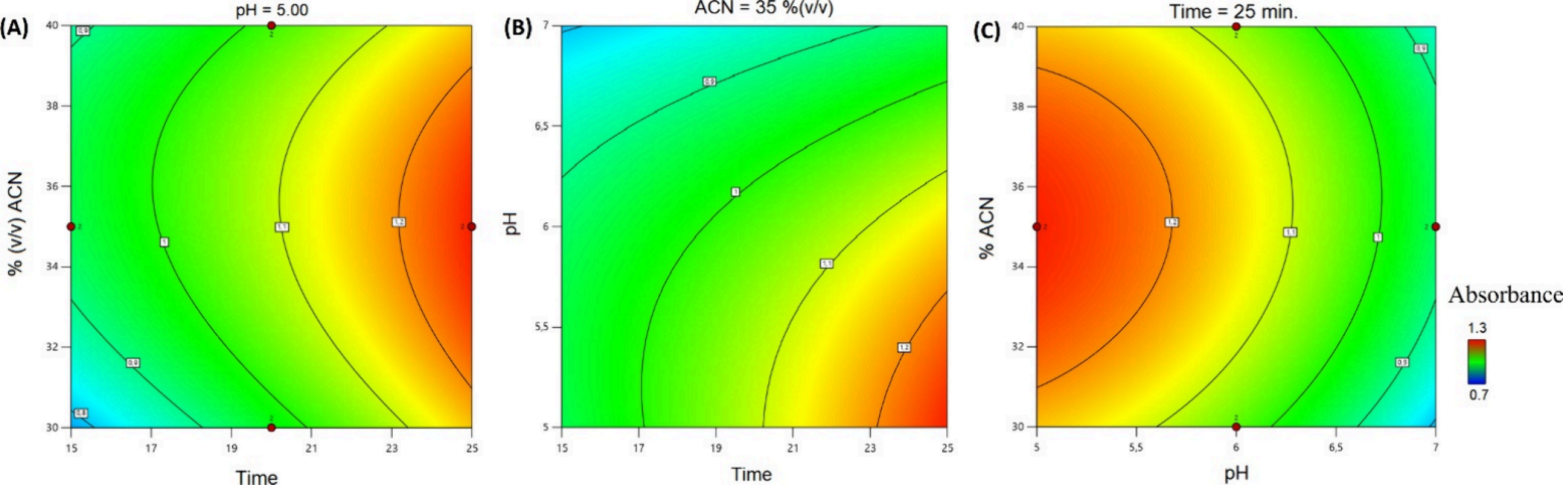
Response surfaces for Box-Behnken design. Evaluation of the interaction between (A) time and % of ACN in the donor phase, (B) donor phase pH and extraction time, and (C) pH % of ACN both from the donor phase. Conditions: electric potential difference of 300 V; donor phase composed of tap water spiked with MB at 0.5 mg L^–1^ and McIlvaine buffer; acceptor phase 0.50 mol L^–1^ acetic acid solution in 0.0300 g of cotton wool. The color scale from blue to red represents the increase in absorbance.

The goodness of fit of the model was evaluated using ANOVA, and the model exhibited no lack of fit at a 95% confidence level, with a *p*-value >0.05. The regression was found to be statistically significant, with a *p*-value <0.05 and the model explaining 98.55% of the variance. The estimated coefficients indicate the expected change in the response when all other factors are held constant. It can be observed that both time and the percentage of ACN had positive coefficients, signifying that higher levels of these variables result in an increase in the response. In contrast, pH displayed a negative coefficient, indicating that the optimal response is achieved at a lower pH level. The interactions among the investigated factors are illustrated by the response surfaces in Figure 3.

When examining the response surfaces, it becomes evident that higher absorbance values are observed when the time approaches the upper limit (25 minutes) and when ACN concentrations are close to the lower limit (35%). Conversely, for pH, an increase in the response is observed when it approaches lower levels (pH = 5.00).

The duration for which the electric potential is applied also plays a crucial role in determining the total number of ions exchanged between the donor and acceptor phases. However, there exists a time restriction for achieving maximum extraction, and this restriction may arise when the constituent ions of the electrolyte solution in the acceptor phase are depleted.^32^ The positive effect on response with the use of ACN in the donor phase has already been mentioned, and it was attributed to the likely effect of electric field increase and interfacial tension reduction.

For MB to undergo electromigration toward the cathode, it must carry a positive charge, which necessitates maintaining the pH of the donor phase at a level that ensures protonation of the dye. This elucidates the reason for the optimal pH being set at 5.00. Under this condition, the dye acquires two positive charges, thus making its extraction mostly governed by electromigration. As pH values exceed 6.00, the molecule begins to shift toward a predominantly electrically neutral state, resulting in reduced electromigration-driven migration (see Figure S3). Based on the results obtained, the optimal conditions for the developed method entail a donor phase comprising McIlvaine buffer at pH = 5.00 with 35% (v/v) ACN and an electroextraction time of 25 min.

### 3.2 Validation Parameters

To ensure the suitability of MPEE for extracting MB from tap water, various method characteristics were evaluated, and the results are summarized in Table 3 and calibration curves shown in Figures S4 and S5. Both precision and accuracy yielded satisfactory outcomes, while the LOD and LOQ values were found to be in line with those reported in other studies in the literature (Table S4). To assess the matrix effect, comparisons were made between the curves constructed using the desorption solvent and those in spiked tap water, utilizing *F*- and *t* tests, both at a 95% confidence level (Figure S6). The statistical tests revealed that the matrix effect was not statistically significant (*p* > 0.05).

**Table 3:** Validation Parameters Evaluated after Factorial Design Optimization

slope (*a*)	intercept	linearity (*r*)	LOQ (mg L^–1^)	LOD (mg L^–1^)
1.8707	0.004	0.998	0.004	0.001

	**sample concentration**		
	0.030 mg L^–1^	0.225 mg L^–1^	0.375 mg L^–1^
intraday precision (RSD, %)	8.6	4.9	3.0
interday precision (RSD, %)	9.9	8.8	9.2

	**sample concentration**		
	0.030 mg L^–1^	0.225 mg L^–1^	0.375 mg L^–1^
average	0.031	0.22	0.39
SD	0.003	0.01	0.01
RSD (%)	8.6	4.9	3.0
recovery (%)	102	99	105

The selectivity of electroextraction techniques relies on the electromigration process, which exclusively affects nonelectrically neutral species with an opposite charge to the acceptor phase electrode. This selectivity, along with its suitability for partitioning into the organic filter, has been previously demonstrated in other studies.^18,19,22^ Furthermore, the stable electric current profile monitored during the extraction process (Figure S7) confirms the successful development of the electroextraction method.

### 3.3 Extraction Efficiency and Preconcentration Factor

After completing the electroextraction of the six spiked samples at 0.030 mg L^–1^, the concentrations of the desorption solutions were determined and the preconcentration factor (PF) for each sample was calculated based on eq S5 (Figure S2), and the results are shown in Table 4.

**Table 4:** Extraction Efficiency (EE) and Preconcentration Factor (PF) of MB in Spiked Tap Water under Optimized Conditions

PF	SD	RSD (%)
10.3	0.9	8.7

EE (%)	SD	RSD (%)
31	2	4.8

The obtained PF was 10.3 (SD = 0.9, *n* = 6), indicating that sample preconcentration up to 10 times was achievable. An advantageous and differential feature of the developed electroextraction method is its capability to accommodate and extract larger sample volumes compared to those typically employed in the literature. Consequently, it becomes feasible to work with samples featuring lower analyte concentrations while achieving a higher preconcentration factor.

### 3.4 SERS Analysis

**Figure 4:**
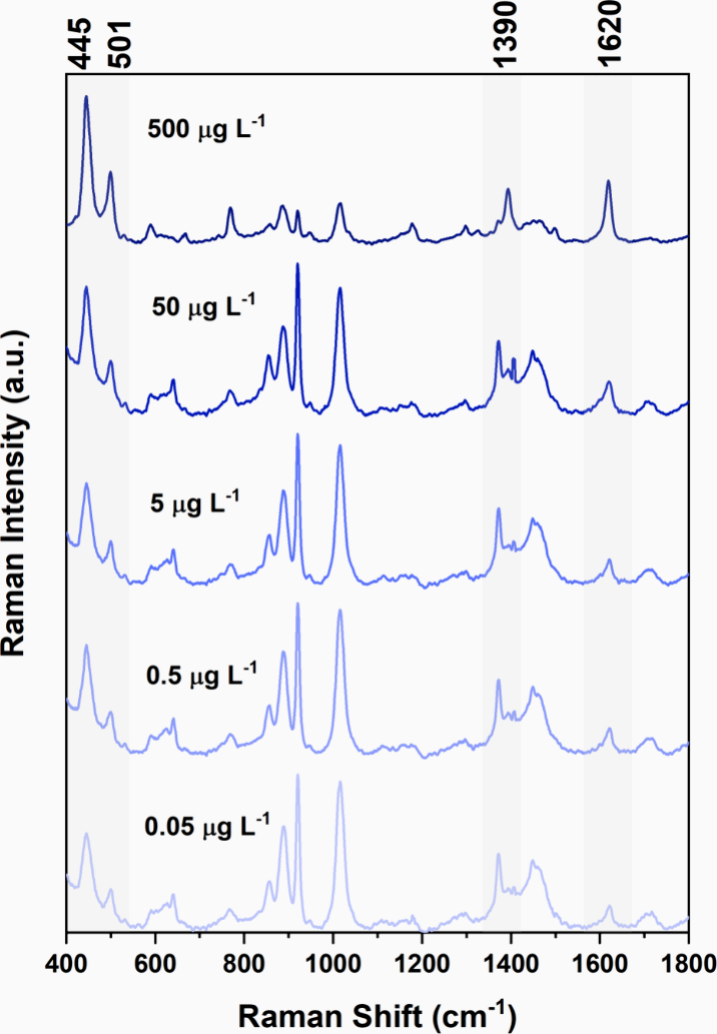
SERS spectra of MB in desorption solution with the respective concentration values in mg L^–1^, referring to the samples before electroextraction. The shadow areas represent MB bands. Excitation laser at 785 nm.

The SERS spectra, obtained from desorbed solutions following the electroextraction of five tap water samples spiked with MB (ranging from 500 to 0.05 μg L^–1^), are presented in Figure 4. To ensure proper desorption of MB from the cotton wool, a solvent mixture (methanol:acetonitrile:acetic acid) was employed, resulting in observable solvent-related signals within the SERS spectra.

However, the distinctive MB bands are readily discernible. These include the prominent band at 1620 cm^–1^, attributed to the C–C ring stretching, followed by the band at 1390 cm^–1^, indicative of symmetric C–N stretching. Additional MB bands appear at 445 and 501 cm^–1^, corresponding to the deformation of the C–N–C skeleton. It is notable that the intensity of these bands decreases as the concentration of MB decreases. Furthermore, regions around 800, 1150, and 1300 cm^–1^ exhibit bands related to MB, although with contributions from solvent due to their association with C–H vibration modes.

SERS offers high sensitivity; however, the technique may lack selectivity toward the target analyte, as any molecule near to or adsorbed on the substrate may experience intensified Raman signals. Therefore, the synergy of electroextraction with SERS holds promise. By combining the substantial preconcentration capabilities of electroextraction with the signal-enhancement properties of SERS, it creates a system that offers both sensitivity and selectivity.

## 4 Conclusions

A newly developed MPEE approach by using an exceptionally cost-effective and readily available sorbent (cottonwood) in conjunction with two distinct, low-cost, and robust spectroscopy analytical techniques (molecular absorption and SERS) was presented. Employing the design of experiments (fractional factorial and Box-Behnken), we swiftly and consistently optimized three extraction parameters (pH, % of acetonitrile in the donor phase, and time), resulting in an efficient, high-throughput sample preparation method capable of processing 10 samples in just 25 min. The stability of the MPEE setup was affirmed by the consistent profile of electric current measurements during extraction. Furthermore, the quality of the optimized extraction method, in tandem with molecular absorption spectroscopy, was validated across all assessed parameters. With an impressive preconcentration factor of 10-fold, this method, when combined with SERS, demonstrates remarkable potential for detecting MB at concentrations below 0.05 μg L^–1^. The methodology developed by combining electroextraction and SERS proves to be highly promising for the detection of the cationic dye in water intended for human consumption. As a perspective, including solid support in MPEE would serve as a notable advantage, allowing direct reading on the Raman spectrometer after adding the SERS substrate. This would eliminate the need for desorption solvents, resulting in cost savings and enhanced process efficiency.

## Supporting Information Available

Description of the synthesis and characterization of the AuNPs; Figure S1: absorption spectrum of the AuNP with the maximum absorption at 520 nm; Figure S2: equations used to calculate some validation parameters; Figure S3: structures and ionization of MB at different pH values; Figure S4: extracted analytical curve (curve 1); Figure S5: extracted analytical curve (curve 2); Figure S6: comparison of extracted analytical curves for matrix effect assessment; Figure S7: current profile for the optimized system; Table S1: design matrix of 2^6–3^ fractional factorial design; Table S2: design matrix of Box-Behnken factorial design; Table S3: *p*-value and estimated coefficients of determination for the adjusted mathematical model; Table S4: works described in the literature that determine the dye MB in aqueous matrices (PDF)

## References

[ref1] Tang Y.; Yin M.; Yang W.; Li H.; Zhong Y.; Mo L.; Liang Y.; Ma X.; Sun X. Emerging pollutants in water environment: Occurrence, monitoring, fate, and risk assessment. Water Environment Research 2019, 91, 984–991. 10.1002/wer.1163. 31220374

[ref2] Schreiner V. C.; Link M.; Kunz S.; Szöcs E.; Scharmüller A.; Vogler B.; Beck B.; Battes K. P.; Cimpean M.; Singer H. P.; Hollender J.; Schäfer R. B. Paradise lost? Pesticide pollution in a European region with considerable amount of traditional agriculture. Water Res. 2021, 188, 116528 10.1016/j.watres.2020.116528. 33126003

[ref3] Puri M.; Gandhi K.; Kumar M. S. Emerging environmental contaminants: A global perspective on policies and regulations. Journal of Environmental Management 2023, 332, 117344 10.1016/j.jenvman.2023.117344. 36736081

[ref4] Pérez-Fernández V.; Mainero Rocca L.; Tomai P.; Fanali S.; Gentili A. Recent advancements and future trends in environmental analysis: Sample preparation, liquid chromatography and mass spectrometry. Anal. Chim. Acta 2017, 983, 9–41. 10.1016/j.aca.2017.06.029. 28811032

[ref5] Ong T. T. X.; Blanch E. W.; Jones O. A. H. Surface Enhanced Raman Spectroscopy in environmental analysis, monitoring and assessment. Sci. Total Environ. 2020, 720, 137601 10.1016/j.scitotenv.2020.137601. 32145632

[ref6] Li X.; Choy W. C. H.; Ren X.; Zhang D.; Lu H. Highly intensified surface enhanced raman scattering by using monolayer graphene as the nanospacer of metal film-metal nanoparticle coupling system. Adv. Funct. Mater. 2014, 24, 3114–3122. 10.1002/adfm.201303384.

[ref7] Lee H. K.; Lee Y. H.; Koh C. S. L.; Phan-Quang G. C.; Han X.; Lay C. L.; Sim H. Y. F.; Kao Y. C.; An Q.; Ling X. Y. Designing surface-enhanced Raman scattering (SERS) platforms beyond hotspot engineering: Emerging opportunities in analyte manipulations and hybrid materials. Chem. Soc. Rev. 2019, 48, 731–756. 10.1039/C7CS00786H. 30475351

[ref8] Huang S.; Fan M.; Wawryk N.; Qiu J.; Yang X.; Zhu F.; Ouyang G.; Li X. F. Recent advances in sampling and sample preparation for effect-directed environmental analysis. TrAC - Trends in Analytical Chemistry 2022, 154, 116654 10.1016/j.trac.2022.116654.

[ref9] Xu J.; Peng V.; Chu J.; Shi J.; Cui Q.; Shi Q. D-limonene as an alternative for the extraction and purification of nuciferine from lotus leaf via multi-stage vortex assisted two-phase solvent extraction integrated with solid phase extraction using mesoporous material SBA-15 as adsorbent. Sustainable Chemistry and Pharmacy 2023, 32, 100997 10.1016/j.scp.2023.100997.

[ref10] García-Valverde M. T.; Soriano M. L.; Lucena R.; Cárdenas S. Cotton fibers functionalized with β-cyclodextrins as selectivity enhancer for the direct infusion mass spectrometric determination of cocaine and methamphetamine in saliva samples. Anal. Chim. Acta 2020, 1126, 133–143. 10.1016/j.aca.2020.05.070. 32736717

[ref11] Lima N. S. M.; Gomes-Pepe E. S.; Kock F. V. C.; Colnago L. A.; de Macedo Lemos E. G. Dynamics of the role of LacMeta laccase in the complete degradation and detoxification of malachite green. World J. Microbiol. Biotechnol. 2023, 39, 127 10.1007/s11274-023-03572-w. 36941452

[ref12] Hansen F. A.; Pedersen-Bjergaard S. Emerging Extraction Strategies in Analytical Chemistry. Anal. Chem. 2020, 92, 2–15. 10.1021/acs.analchem.9b04677. 31625733

[ref13] Pedersen-Bjergaard S.; Rasmussen K. E. Electrokinetic migration across artificial liquid membranes: New concept for rapid sample preparation of biological fluids. Journal of Chromatography A 2006, 1109 (2), 183–190. 10.1016/j.chroma.2006.01.025. 16445928

[ref14] He Y.; Miggiels P.; Wouters B.; Drouin N.; Guled F.; Hankemeier T.; Lindenburg P. W. A high-throughput, ultrafast, and online three-phase electro-extraction method for analysis of trace level pharmaceuticals. Anal. Chim. Acta 2021, 1149, 338204 10.1016/j.aca.2021.338204. 33551054

[ref15] Wu J.; Huang X. Electric field-reinforced solid phase microextraction based on anion-exchange monolith for efficient entrapment of anions in aqueous and wine samples. Journal of Chromatography A 2022, 1676, 463291 10.1016/j.chroma.2022.463291. 35792441

[ref16] Román-Hidalgo C.; Barreiros L.; Villar-Navarro M.; López-Pérez G.; Martín-Valero M. J.; Segundo M. A. Electromembrane extraction based on biodegradable materials: Biopolymers as sustainable alternatives to plastics. TrAC - Trends in Analytical Chemistry 2023, 162, 117048 10.1016/j.trac.2023.117048.

[ref17] Yuan J.; Cao H.; Du X.; Chen T.; Ma A.; Pan J. Nonaqueous miscible liquid-liquid electroextraction for fast exhaustive enrichment of ultratrace analytes by an exponential transfer and deceleration mechanism. Anal. Chem. 2021, 93 (3), 1458–1465. 10.1021/acs.analchem.0c03478. 33375784

[ref18] Orlando R. M.; Nascentes C. C.; Botelho B. G.; Moreira J. S.; Costa K. A.; de Miranda Boratto V. H. Development and Evaluation of a 66-Well Plate Using a Porous Sorbent in a Four-Phase Extraction Assisted by Electric Field Approach. Anal. Chem. 2019, 91 (10), 6471–6478. 10.1021/acs.analchem.8b04943. 31074962

[ref19] Sousa D. V. M; Pereira F. V.; Nascentes C. C.; Moreira J. S.; Boratto V. H. M.; Orlando R. M. Cellulose cone tip as a sorbent material for multiphase electrical field-assisted extraction of cocaine from saliva and determination by LC-MS/MS. Talanta 2020, 208, 120353 10.1016/j.talanta.2019.120353. 31816720

[ref20] Viana J. d. S.; Caneschi de Freitas M.; Botelho B. G.; Orlando R. M. Large-volume electric field-assisted multiphase extraction of malachite green from water samples: A multisample device and method validation. Talanta 2021, 222, 121540 10.1016/j.talanta.2020.121540. 33167248

[ref21] Sousa D. V. M.; Pereira F. V.; Boratto V. H. M.; Orlando R. M. Multiphase electroextraction as a simple and fast sample preparation alternative for the digital image determination of doxorubicin in saliva. Talanta 2023, 255, 124242 10.1016/j.talanta.2022.124242. 36638654

[ref22] Ferreira Avelar M. C.; Nascentes C. C.; Orlando R. M. Electric field-assisted multiphase extraction to increase selectivity and sensitivity in liquid chromatography-mass spectrometry and paper spray mass spectrometry. Talanta 2021, 224, 121887 10.1016/j.talanta.2020.121887. 33379096

[ref23] Amador V. S.; Moreira J. S.; Augusti R.; Orlando R. M.; Piccin E. Direct coupling of paper spray mass spectrometry and four-phase electroextraction sample preparation. Analyst 2021, 146, 1057–1064. 10.1039/D0AN01699C. 33331369

[ref24] Rathi B. S.; Kumar P. S.; Vo D. V. N. Critical review on hazardous pollutants in water environment: Occurrence, monitoring, fate, removal technologies and risk assessment. Sci. Total Environ. 2021, 797, 149134 10.1016/j.scitotenv.2021.149134. 34346357

[ref25] Tkaczyk A.; Mitrowska K.; Posyniak A. Synthetic organic dyes as contaminants of the aquatic environment and their implications for ecosystems: A review. Sci. Total Environ. 2020, 717, 137222 10.1016/j.scitotenv.2020.137222. 32084689

[ref26] Mcilvaine T. C. A buffer solution for colorimetric comparison. J. Biol. Chem. 1921, 49 (1), 183–186. 10.1016/S0021-9258(18)86000-8.

[ref27] Alves I. M.; Melo N. O.; Marinho P. A.; Almeida M. R. Liquid-liquid extraction-assisted SERS-based detection of clonazepam in spiked drinks. Vib. Spectrosc. 2020, 110, 103112 10.1016/j.vibspec.2020.103112.

[ref28] Raterink R. J.; Lindenburg P. W.; Vreeken R. J.; Hankemeier T. Three-phase electroextraction: A new (Online) sample purification and enrichment method for bioanalysis. Anal. Chem. 2013, 85 (16), 7762–8. 10.1021/ac4010716. 23724849

[ref29] Seip K. F.; Jensen H.; Sønsteby M. H.; Gjelstad A.; Pedersen-Bjergaard S. Electromembrane extraction: Distribution or electrophoresis?. Electrophoresis 2013, 34 (5), 792–799. 10.1002/elps.201200587. 23255056

[ref30] Restan M. S.; Jensen H.; Shen X.; Huang C.; Martinsen Ø.G.; Kubáň P.; Gjelstad A.; Pedersen-Bjergaard S. Comprehensive study of buffer systems and local pH effects in electromembrane extraction. Anal. Chim. Acta 2017, 984, 116–123. 10.1016/j.aca.2017.06.049. 28843554

[ref31] Seip K. F.; Jensen H.; Kieu T. E.; Gjelstad A.; Pedersen-Bjergaard S. Salt effects in electromembrane extraction. Journal of Chromatography A 2014, 1347, 1–7. 10.1016/j.chroma.2014.04.053. 24792700

[ref32] Nojavan S.; Tahmasebi Z.; Bidarmanesh T.; Behdad H.; Nasiri-Aghdam M.; Mansori S.; Pourahadi A. Electrically enhanced liquid-phase microextraction of three textile azo dyes from wastewater and plant samples. J. Sep. Sci. 2013, 36 (19), 3256–63. 10.1002/jssc.201300546. 23894042

