# Supplementary material for: Enhancing Analytical Sensitivity and Selectivity for Methylene Blue Determination in Water Samples by Using Multiphase Electroextraction Coupled with Optical Absorption Spectroscopy and Surface-Enhanced Raman Scattering
Source: ACS Omega. 2024 Jul 20;9(30):32769–76. doi: 10.1021/acsomega.4c03125 (PMC11292653; doi:10.1021/acsomega.4c03125)
Supplement: Supplementary file 1 — ao4c03125_si_001.pdf [file ao4c03125_si_001.pdf]

SUPPORTING INFORMATION

Enhancing Analytical Sensitivity and Selectivity for Methylene Blue Determination in Water Samples by Using Multiphase Electroextraction Coupled with Optical Absorption Spectroscopy and Surface-Enhanced Raman Scattering

Tarlene P. Miranda[a], Ricardo M. Orlando*[ab], Cristiano Fantini[c], Mariana R. Almeida*[a]

[a] Departamento de Química, Instituto de Ciências Exatas, Universidade Federal de Minas Gerais, UFMG, 31270-901, Belo Horizonte, MG, Brazil

[b] Laboratório de Microfluídica e Separações, LaMS, Departamento de Química, Universidade Federal de Minas Gerais, Belo Horizonte, 30123-970, Minas Gerais, Brazil

[c] Departamento de Física, Instituto de Ciências Exatas, Universidade Federal de Minas Gerais, UFMG, 31270-901, Belo Horizonte, MG, Brazil

Synthesis and characterization of gold nanoparticles

To prepare the nanospheres, 95.00 mL of deionized water (Milli-Q system) and 36.00 µL of tetrachloroauric acid ($HAuCl_4$, Sigma-Aldrich) at 30% m/m were added to an Erlenmeyer flask protected from light, keeping the solution under stirring at 2000 rpm and heating until boiling. After boiling the solution, dropwise, 3.00 mL of a heated solution of sodium citrate ($Na_3C_6H_5O_7$, Sigma-Aldrich) at 1% m/m was added. After the solution changed color from colorless to purple (approximately 10 minutes), heating and stirring were turned off. The solution was stored in an amber bottle under refrigeration until use. The nanoparticle formation was confirmed by the visible absorption spectrum with the plasmonic band of gold at 520 nm, as shown in Figure S1.

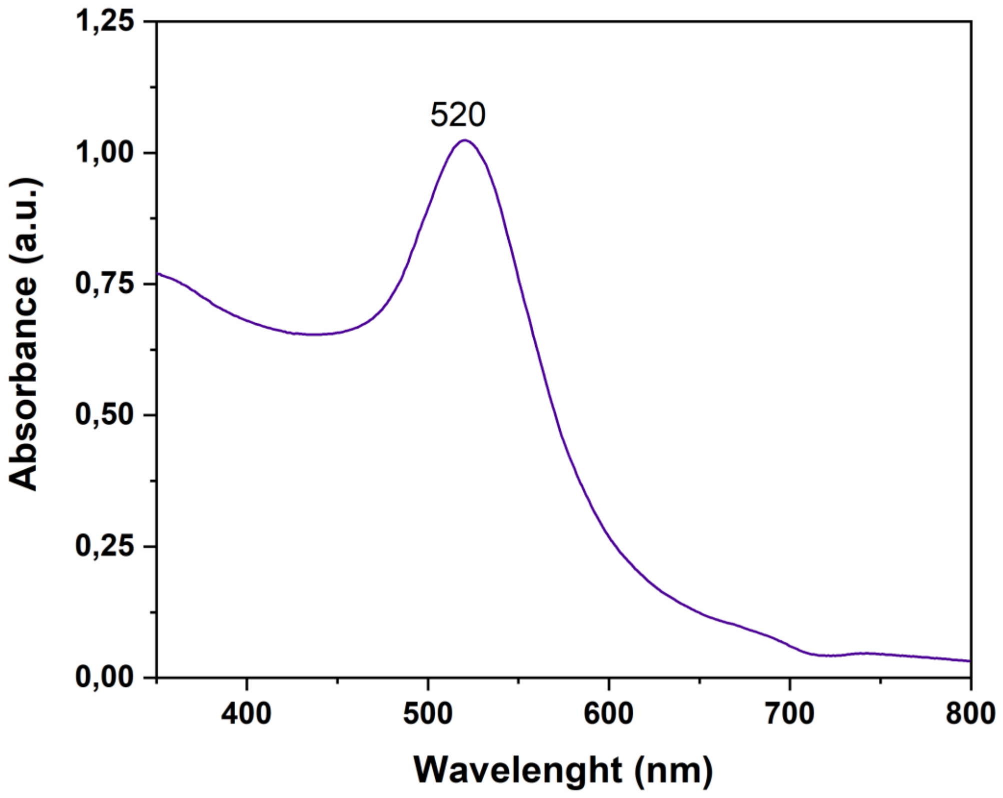

Figure S1: Absorption spectrum of the AuNP with the maximum of absorption at 520 nm.

$$Recovery\ (\%) = \frac{Calculated\ concentration \cdot 100}{Theoretical\ concentration} \quad \textbf{(eq. 1)}$$

$$(\%)EE = \frac{\left(\frac{Absorbance\ of\ analyte\ in\ extract}{FC}\right) \cdot 100}{Average\ absorbance\ of\ analyte\ in\ sample} \quad \textbf{(eq. 2)}$$

$$LOQ = \frac{10 \cdot s}{a} \quad \textbf{(eq. 3)}$$

$$LOD = \frac{3.3 \cdot s}{a} \quad \textbf{(eq. 4)}$$

$$PF = \frac{C_{ap}}{C_{dp}} \quad \textbf{(eq. 5)}$$

Fig. S2. Equations used to calculate $Recovery$, extraction efficiency (EE), dilution factor (DF), limit of detection (LOD), limit of quantitation (LOQ) and pre-concentration factor (PF). s = standard deviation of extracted blank samples; C_{ap}= concentration of analyte in acceptor phase; C_{dp} = concentration of analyte in the donor phase.

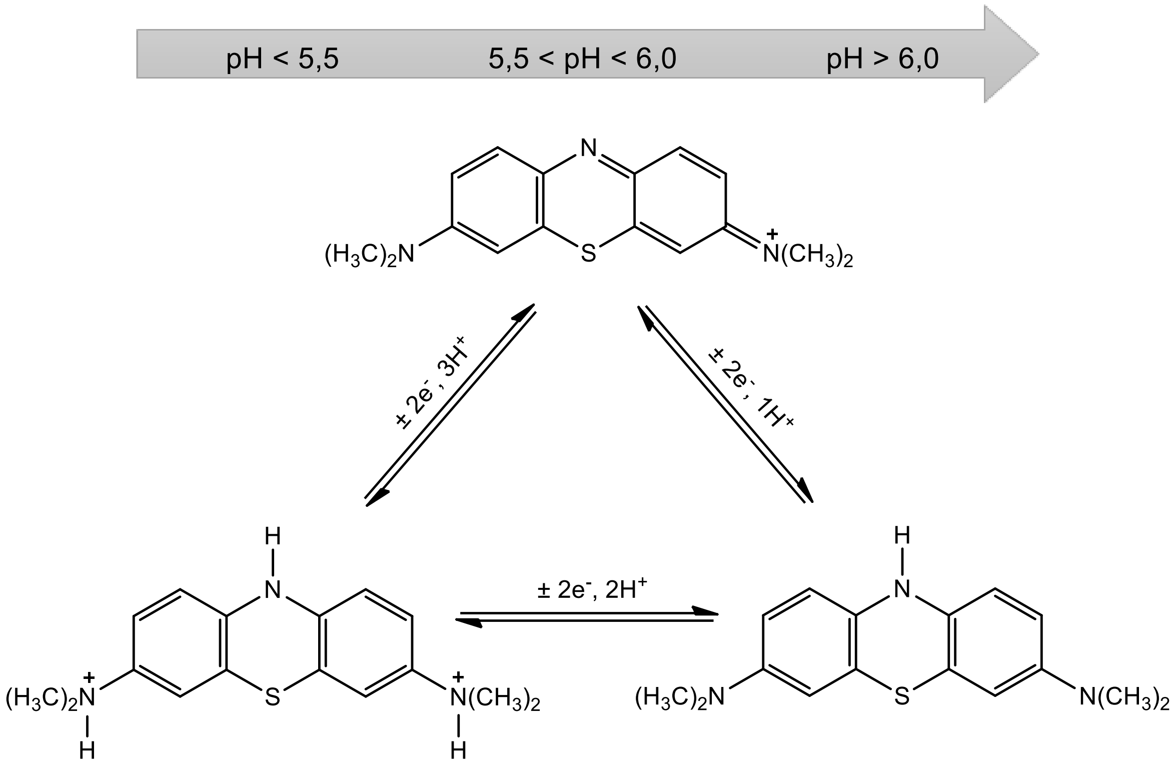

Figure S3. Structures and ionization of MB at different pH values. Adapted from [1].

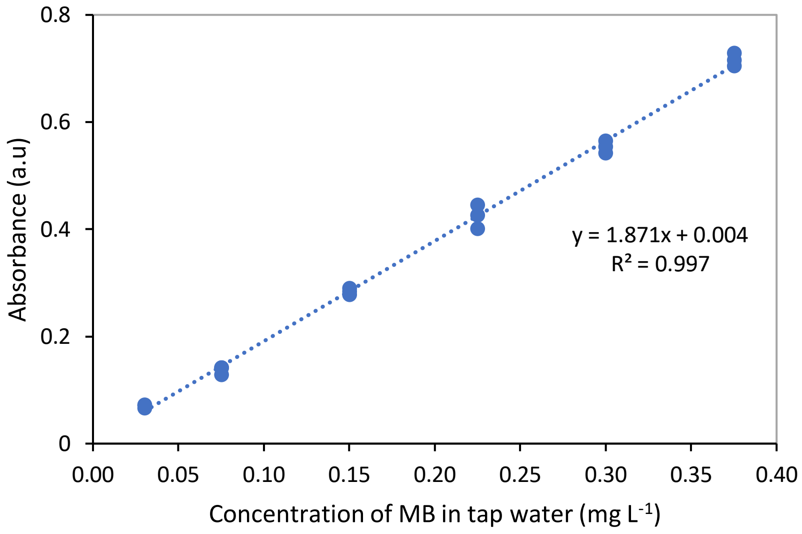

Figure S4. Extracted analytical curve (curve 1) for spiked tap water with MB. Absorbance at 654 nm.

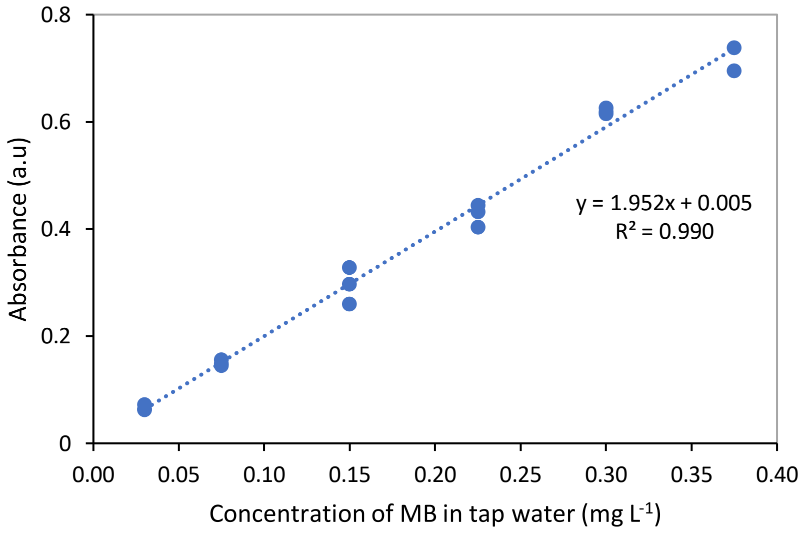

Figure S5. Extracted analytical curve (curve 2) for spiked tap water with MB. Absorbance at 654 nm.

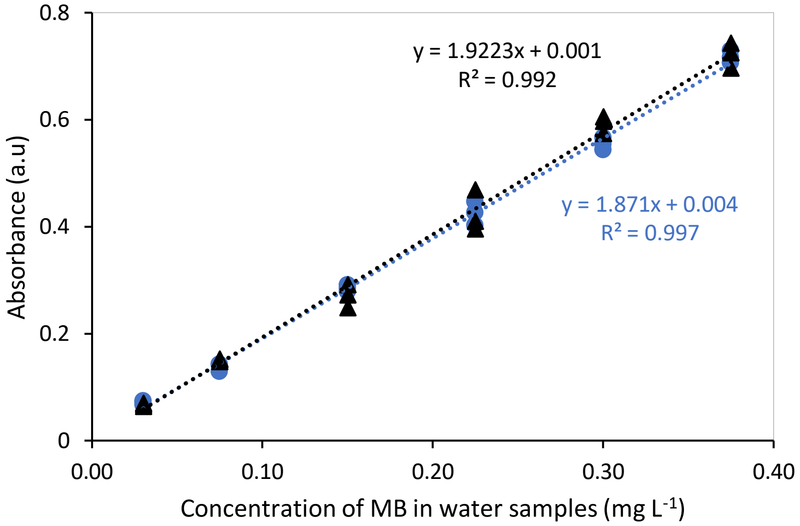

Figure S6. Comparison of extracted analytical curves in deionized water (blue circles) and tap water (black triangles) for matrix effect assessment. Absorbance at 654 nm.

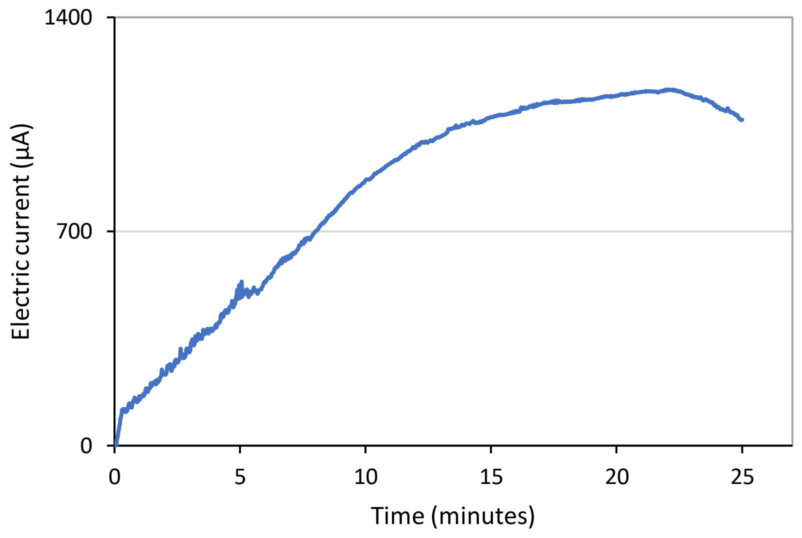

Figure S7. Current profile for the optimized system. Conditions: 32 mL of donor phase (tap water:McIlvaine buffer); organic filter consisting of 3.00 mL of 1-octanol; acceptor phase comprised of 0.50 mol L^{-1} acetic acid immobilized on 0.0300 g of cotton wool; and an applied electric potential difference of 300 V for 25 minutes.

Table S1. Design matrix of 2^{6-3} fractional factorial design.

Exp[a]	Type of acceptor phase electrolyte	Donor phase pH	Type of organic solvent in the donor phase	Extraction time (min)	Sorbent amount in the acceptor phase (g)	Type of organic filter
**1**	HAc	2.00	ETOH	10	0.0300	1-octanol
**2**	HCl	2.00	ETOH	20	0.0150	2-ethylhexanol
**3**	HAc	7.00	ETOH	20	0.0150	1-octanol
**4**	HCl	7.00	ETOH	10	0.0300	2-ethylhexanol
**5**	HAc	2.00	ACN	20	0.0300	2-ethylhexanol
**6**	HCl	2.00	ACN	10	0.0150	1-octanol
**7**	HAc	7.00	ACN	10	0.0150	2-ethylhexanol
**8**	HCl	7.00	ACN	20	0.0300	1-octanol

[a] = experiment.

Table S2. Design matrix of Box-Behnken factorial design.

Experiment	Extraction time (min)	Donor phase pH	% (v/v) of organic solvent (ACN) in the donor phase
**1**	15	5.00	35
**2**	25	5.00	35
**3**	15	7.00	35
**4**	25	7.00	35
**5**	15	6.00	30
**6**	25	6.00	30
**7**	15	6.00	40
**8**	25	6.00	40
**9**	20	5.00	30
**10**	20	7.00	30
**11**	20	5.00	40
**12**	20	7.00	40
**PC**	20	6.00	35
**PC**	20	6.00	35
**PC**	20	6.00	35

Table S3. p-value and estimated coefficients of determination for the adjusted mathematical model.

Parameters	p-value for lack of fit	Coefficients of determination
**A - time**	< 0.0001	0.1163
**B - pH**	< 0.0001	-0.1196
**C - % ACN**	< 0.0001	0.0439
**AB**	< 0.0001	- 0.0496
**AC**	0.0033	- 0.0270
**BC**	0.0383	0.0178
**A^2**	0.5137	0.0063
**B^2**	< 0.0001	- 0.0564
**C^2**	< 0.0001	- 0.1003
**Lack of fit**	0.2259	-
**Model**	< 0.0001	-
**Explained variation (R^2) = 0.9855**		

Table S4. Works described in the literature that determine the dye MB in aqueous matrices.

Water sample	Sample preparation	Analytical technique	LOD (mg L^{-1})	LOQ (mg L^{-1})	Linear range (mg L^{-1})	Ref
**Triple-distilled water**	-	SERRS	7.47×10^{-5}	2.27×10^{-4}	$1.60 \times 10^{-5} – 4.50 \times 10^{-2}$	[1]
**Triple-distilled water**	-	EAM-UV/Vis	0.19	0.58	0.20 – 6.80	[1]
**Wastewater**	SALLE	EAM-UV/Vis	0.20	0.06	0.20 – 7.00	[2]
**Industrial effluent**	SPE	UPLC-MS/MS	1.00×10^{-4}	4.00×10^{-4}	$1.00 \times 10^{-5} – 5.00 \times 10^{-3}$	[3]
**Wastewater, river water, rainwater, tap water, lake water**	DLLME	EAM-UV/Vis	7.90×10^{-4}	2.60×10^{-3}	5.00×10^{-3} – 3.00	[4]
**Wastewater, river water, bottled water**	MSPE-DLLME	HPLC-UV	$5.00\text{x } 10^{-5}$	1.60×10^{-4}	$2.00 \times 10^{-4} – 1.00 \times 10^{-2}$	[5]
**Domestic wastewater, river water**	DSPE-CPE	EAM-UV/Vis	6.50×10^{-4}	2.05×10^{-3}	$2.00 \times 10^{-3} – 3.00 \times 10^{-1}$	[6]

CPE: cloud-point extraction; DLLME: dispersive liquid-liquid microextraction; DSPE: dispersive solid-phase extraction; UV/Vis: UV/visible molecular absorption spectrophotometry; HPLC-UV: high-performance liquid chromatography with UV detector; LOD: limit of detection; LOQ: limit of quantification; MSPE: magnetic solid-phase extraction; Ref: reference; SALLE: salt-assisted liquid-liquid extraction; SERRS: surface-enhanced resonance raman scattering; SPE: solid-phase extraction; UPLC-MS/MS: ultra-high-performance liquid chromatography coupled with tandem mass spectrometry.

References

[1] Anastasopoulos JA, Soto Beobide A, Manikas AC, Voyiatzis GA. Quantitative surface-enhanced resonance Raman scattering analysis of methylene blue using silver colloid. J Raman Spectrosc. 2017;48(12):1762–70.

[2] Razmara RS, Daneshfar A, Sahrai R. Determination of methylene blue and sunset yellow in wastewater and food samples using salting-out assisted liquid-liquid extraction. J Ind Eng Chem . 2011;17(3):533–6.

[3] Khan MR, Khan MA, Alothman ZA, Alsohaimi IH, Naushad M, Al-Shaalan NH. Quantitative determination of methylene blue in environmental samples by solid-phase extraction and ultra-performance liquid chromatography-tandem mass spectrometry: A green approach. RSC Adv. 2014;4(64):34037–44.

[4] Asfaram A, Ghaedi M. Simultaneous determination of cationic dyes in water samples with dispersive liquid-liquid microextraction followed by spectrophotometry: Experimental design methodology. New J Chem. 2016;40(5):4793–802.

[5] Liang N, Hou X, Huang P, Jiang C, Chen L, Zhao L. Ionic liquid-based dispersive liquid-liquid microextraction combined with functionalized magnetic nanoparticle solid-phase extraction for determination of industrial dyes in water. Sci Rep. 2017;7(1):1–9.

[6] Nekouei F, Kargarzadeh H, Nekouei S, Keshtpour F, Makhlouf ASH. Efficient method for determination of methylene blue dye in water samples based on a combined dispersive solid phase and cloud point extraction using $Cu(OH)_2$ nanoflakes: central composite design optimization. Anal Bioanal Chem. 2017;409(4):1079–92.
